# Microextraction by Packed Sorbent (MEPS) and Solid-Phase Microextraction (SPME) as Sample Preparation Procedures for the Metabolomic Profiling of Urine

**DOI:** 10.3390/metabo4010071

**Published:** 2014-01-27

**Authors:** Catarina Silva, Carina Cavaco, Rosa Perestrelo, Jorge Pereira, José S. Câmara

**Affiliations:** 1 CQM—Centro de Química da Madeira, Universidade da Madeira, Campus Universitário da Penteada, Funchal 9000-390, Portugal; E-Mails: cgsluis@uma.pt (C.S.); carina-cavaco@hotmail.com (C.C.); rmp@uma.pt (R.P.); jamp@uma.pt (J.P.); 2 Centro de Ciências Exatas e da Engenharia da Universidade da Madeira, Campus Universitário da Penteada, Funchal 9000-390, Portugal

**Keywords:** urine, metabolomics, microextraction by packed sorbent, solid-phase microextraction

## Abstract

For a long time, sample preparation was unrecognized as a critical issue in the analytical methodology, thus limiting the performance that could be achieved. However, the improvement of microextraction techniques, particularly microextraction by packed sorbent (MEPS) and solid-phase microextraction (SPME), completely modified this scenario by introducing unprecedented control over this process. Urine is a biological fluid that is very interesting for metabolomics studies, allowing human health and disease characterization in a minimally invasive form. In this manuscript, we will critically review the most relevant and promising works in this field, highlighting how the metabolomic profiling of urine can be an extremely valuable tool for the early diagnosis of highly prevalent diseases, such as cardiovascular, oncologic and neurodegenerative ones.

## 1. Introduction

The development of an analytical method includes several steps, such as sampling and extraction, analysis and, finally, the mathematical processing. All of them greatly influence the analytical performance that can be achieved in terms of reliability, accuracy, precision and sensitivity, as well as the time and cost of analysis. In several cases, over 80% of analysis time is spent on sampling and sample preparation steps, including homogenization, extraction, concentration and clean-up. This is necessary for several matrices, such as the biological ones, once the analytical instruments cannot handle the sample complexity directly. Therefore, sample preparation has been recognized as the main bottleneck of the analytical process, particularly for the analysis of trace components [1,2].

In this sense, an ideal sample preparation should present the following features: (i) minimal sample loss and a maximum recovery of the target analyte; (ii) elimination of coexisting components with a high yield; (iii) a simple, fast and inexpensive method; (iv) compatibility with the following analytical instruments; and (v) in conformity with green chemistry demands [3,4,5,6,7]. Microextraction techniques (METs), which use a minimal extractant amount (sorbent or liquid phase) offer these benefits and are becoming widely used in different fields, such as the biomedical, food, forensic and environmental ones, just to name the most frequently described applications (reviewed in [8,9,10,11,12,13]).

**Figure 1:**
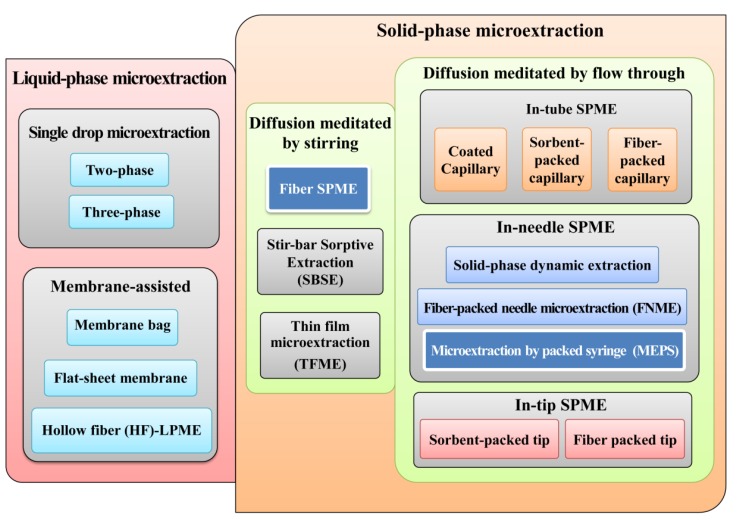
Classification of microextraction techniques (METs).

Abbreviations: FNME, fiber-packed needle microextraction; MEPS, microextraction by packed sorbent; SBSE, stir-bar sorptive extraction; SPDE, solid phase dynamic extraction; SPME, solid-phase microextraction; TFME, thin film microextraction.

The recent advances in this field have converged on the miniaturization and integration of sample preparation online with analytical instrumentation, in order to reduce laboratory workload and to increase analytical performance [14]. From this perspective, METs have emerged in the last few years as powerful sample preparation approaches suitable for easily automating with liquid and gas chromatographic systems applied in a diversity of bioanalytical areas. Nowadays, there are several MET formats available that can be grouped in liquid-phase and solid-phase METs (Figure 1). In the first group, we have the single-drop and the membrane-assisted microextractions variants. The second group, the solid-phase METs, can be organized according to the diffusion process in stirring and flow through versions. Nevertheless, solid-phase microextraction (SPME) in its different formats and microextraction by packed sorbent (MEPS) are certainly two of the most successful METs currently used with an increasing range of applications. Moreover, the aim of this review is to discuss the main advantages of using urine as a biological matrix suitable for diagnostic purposes combined with high throughput analytical techniques. 

## 2. Extraction Techniques

### 2.1. Solid-Phase Microextraction (SPME)

SPME was introduced by Arthur and Pawliszyn in the early 1990s [15], and it is based on the partitioning of target analytes between the sample and the stationary phase, which is typically coated in the surface of a fused silica fiber (1–2 cm). The analytes are then thermally desorbed in a gas chromatography (GC) injector port or removed by solvents for high performance liquid chromatography (HPLC) or electrophoresis applications and subsequently analyzed. This combination allows for an excellent analytical performance for the quantification of different chemical families [16,17,18].

**Figure 2:**
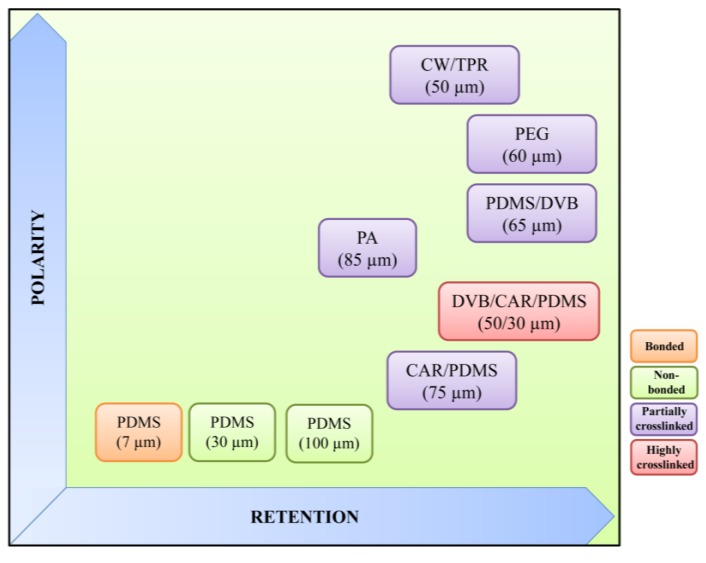
Properties of commercially available SPME fibers (adapted from [31]). CW/TPR, carbowax/templated resin; PEG, polyethylene glycol; PDMS, poly(dimethylsiloxane); DVB, divinylbenzene; PA, polyacrylate; CAR, carboxen.

The main SPME advantages are the simplicity of operation, its solventless nature, analyte/matrix separation and pre-concentration, the availability of different commercial fibers, as well as the developments toward the automation of the whole process that have made SPME a routinely used tool in food, environmental, clinical, pharmaceutical and bioanalysis applications [19,20,21]. Its use in analytical laboratories is, therefore, expected to continue to grow in the future [22]. The generally accepted drawbacks are a relatively poor reproducibility, lot-to-lot variations, the lack of selectivity, sensibility against organic solvents, the latter frequently preventing liquid sampling, and their cost. Nevertheless, possibly the most important disadvantage is the limited range of stationary phases available, only roughly covering the scale of polarity (see Figure 2) [23,24]. Fiber coating procedures, which included sol-gel technology, electrochemical methods and physical deposition, provide a wide range of homemade coatings, which can sort out some of the drawbacks associated with the commercial fibers [24,25].

#### 2.1.1. SPME Technical Aspects and Analytical Performance

##### 2.1.1.1. Extraction Mode

For the SPME extraction, fused silica coated fiber can be introduced into the sample in three different ways: (i) direct extraction; (ii) headspace (HS-SPME); and (iii) extraction with membrane protection. Obviously, there are many factors affecting both of these sampling procedures, and some of these will be discussed in the current manuscript [22,26,27]. The extraction efficiency of each mode depends on the analytes properties and the sample matrix (reviewed in [22]).

(i) Direct extraction (DI-SPME)

The coated fiber is directly immersed in the aqueous samples, and the analytes are transported directly from the sample matrix into the extracting phase. The sample agitation is often carried out with a small stirring bar to decrease the time necessary for equilibration time and to improve the analyte transportation from the sample bulk to the fiber vicinity [28,29].

(ii) Headspace (HS-SPME)

In headspace mode, the analytes are extracted from the gas phase above a gaseous, aqueous or solid sample. The primary reason for this modification is to protect the fiber from adverse effects caused by non-volatile, high molecular-weight substances present in the sample matrix (e.g., proteins). The headspace mode also allows for matrix modifications (including pH adjustment) without affecting the fiber. In a system consisting of a liquid sample and its headspace, the amount of an analyte extracted by the fiber coating does not depend on the location of the fiber (in the liquid or gas phase) [28]. The analyte amount sorbed on the fiber, and the resulting sensitivity, are determined both by sorption kinetics and the distribution coefficient of the compound between the coating fiber, the headspace and the sample (reviewed in [30]).

(iii) Extraction with membrane protection

A selective membrane separated the sample from the fiber, which lets the analytes through, while blocking the interferences. The main purpose for the use of the membrane barrier is to protect the fiber against adverse effects caused by high molecular-weight compounds when very complex samples are analyzed [28].

##### 2.1.1.2. Coating Fibers

Several types of stationary phases, of different thicknesses and polarities, are commercially available (Supelco, Gland, Switzerland ), showing great selectivity for different analytes, namely three poly(dimethylsiloxane) (PDMS) films of different thicknesses (seven, 30 and 100 µm), 85 µm polyacrylate (PA), the 60 and 65 µm polydimethylsiloxane/divinylbenzene (PDMS/DVB) mixed phases, 75 µm carboxen/polydimethylsiloxane (CAR/PDMS), 60 µm polyethylene glycol (PEG), 50 µm carbowax/templated resin (CW/TPR) and 50/30 µm divinylbenzene/carboxen/polydimethylsiloxane (DVB/CAR/PDMS). Stationary phases are immobilized by non-bonding, bonding, partial crosslinking or high crosslinking (Figure 2). Non-bonded phases are stable with some water-miscible organic solvents, although some swelling may occur when used with non-polar solvents. Bonded phases are stable with all organic solvents, except for some non-polar solvents. Finally, partially crosslinked phases are stable in most water-miscible organic solvents and some polar solvents, while highly crosslinked phases are similar to the latter, except that some bonding to the core may occur (reviewed in [22]).

Regarding fiber polarity, polar fibers are effective for extracting polar analytes, and nonpolar fibers are effective for extracting the nonpolar analytes from different matrices. Fibers with different polarities provide high extraction selectivities and reduce the possibility of extracting interferences. For example, PDMS/DVB, PDMS/CAR and PEG fiber coatings are more polar than those containing PA, and for this reason, they are more often used to extract highly polar compounds, like alcohols and carboxylic acids; on the other hand, CAR is responsible for the CAR/PDMS coating’s greater specific surface area, and this results in a very efficient extraction of volatile organic compound (VOC) analytes [25].

##### 2.1.1.3. Extraction Time and Temperature

Extraction time and temperature are two of the most important parameters affecting the SPME extraction efficiency. The extraction time is dependent on the partition coefficient of the analyte between the fiber coating and the sample matrix and also on the sample stirring, this being generally shorter for extractions from the headspace. SPME has a maximum sensitivity at the equilibrium point, defined as the time after which the level of analyte extracted remains constant, corresponding to the limit of experimental error to the amount extracted after infinite time. At equilibrium, small extraction time variations do not affect the level of analyte extracted by the fiber. Moreover, full equilibration is not necessary for accurate and precise analysis by SPME, due to the linear relationship between the amount of analyte adsorbed by the SPME fiber and its initial concentration in the sample matrix in non-equilibrium conditions (reviewed in [22,30]).

SPME extraction is an exothermic equilibration process and, therefore, the increase in the extraction temperature causes an increase in the extraction rate, and simultaneously, the distribution constant decreases [28]. On the other hand, the headspace-analyte partition coefficient increases with higher sampling temperature, resulting in a higher analyte concentration in the headspace and a consequent shorter extraction time [22]. In the case of natural product extraction, less aggressive conditions should be applied, such as moderate temperature and protection from light and oxygen, to prevent the degradation of some thermosensitive compounds.

##### 2.1.1.4. Ionic Strength

The salt addition can influence the extraction efficiency by changing the properties of the boundary phase and decreasing the solubility of hydrophilic compounds in the aqueous phase (salting-out effect) [32]. However, the salt addition is preferred for HS-SPME, because fiber coatings are prone to damage during agitation by direct extraction (DI)-SPME. For this purpose, sodium chloride, sodium hydrogen carbonate, potassium carbonate and ammonium sulfate are generally used [22].

### 2.2. Microextraction by Packed Sorbent (MEPS)

MEPS was introduced by Abdel-Rehim in 2004 [11]. It is a miniaturization of the conventional solid-phase extraction (SPE)-packed bed devices from milliliter bed volumes to microliter volumes, which can be connected online to gas chromatography (GC) and/or liquid chromatography (LC) without any further modifications [33]. This technique has been successfully used to extract a wide range of analytes in different biological matrices, such as urine, plasma, saliva and blood [11,34,35].

**Figure 3:**
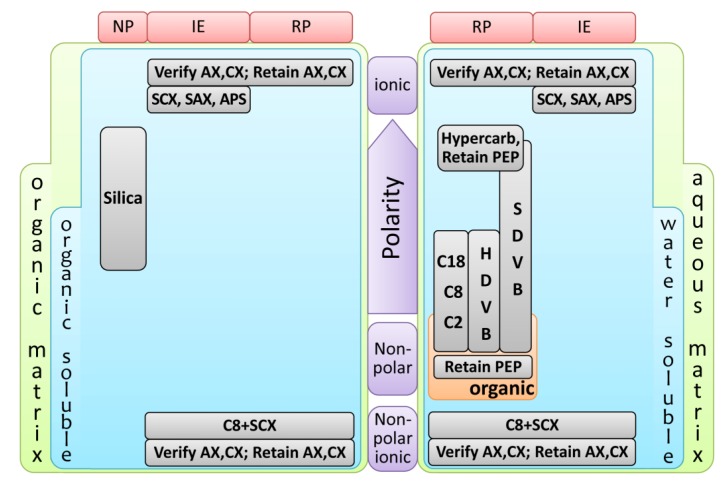
Simplified flowchart for MEPS sorbents selection. The parameters used were the matrix properties (aqueous or organic), the polarity and solubility of the target analytes (soluble in water or organic solvents) and the extraction mode (reverse-phase (RP), ion-exchange (IE) or normal-phase (NP)). Adapted from [9]. APS, AminoPropyl Siloxane; AX, Anion eXchange; CX, Cation eXchange; HDVB, Highly cross-linked polystyrene DiVinylBenzene; PEP, Polar Enhanced Polymer; SAX, Strong Anion eXchange; SCX, Strong Cation eXchange; SDVB, polyStyrene DiVinylBenzene.

In MEPS, approximately 1–2 mg of the sorbent is packed inside a syringe (100–250 µL) as a plug or between the barrel and the needle as a cartridge. Sample extraction takes place in this packed bed, which can be coated to provide selective and suitable sampling conditions. The MEPS approach to sample preparation is suitable for reversed phases (extraction of hydrophobic analytes or polar organic analytes from aqueous matrices), normal phases (extraction of polar analytes from non-polar organic solvents) and mixed mode and ion exchange chemistries (extraction of charged analytes from aqueous or non-polar organic samples) [9,34,35]. There are several available MEPS sorbent materials (Figure 3), including reversed phase (C18, C8 and C2), normal phase (silica), restricted access material (RAM), HILIC (hydrophilic interaction liquid chromatography), carbon, polystyrene-divinylbenzene copolymer (PS-DVB), molecular imprinted polymers (MIPs), strong cation exchange (SCX) and mixed mode (C8/SCX) chemistries (reviewed in [9,35]).

This extraction method differs from commercial solid-phase extraction (SPE) in that the packing is integrated directly into the syringe and not into a separate column. Furthermore, the packed sorbent can be used more than 100 times, even when using plasma or urine samples, but a conventional SPE column is used only once. Moreover, MEPS can handle low sample volumes (10 µL) to large volumes (1,000 µL). The analytes are then eluted with small volumes of an organic solvent, such as methanol or other mobile phases, allowing for a very significant concentration of the target when large sample volumes are used. The combination of MEPS and chromatographic techniques, such as GC-MS, HPLC and LC-MS/MS, is an excellent tool for the screening and determination of biomarkers in biological samples. This approach for sample preparation is therefore very promising for many reasons, namely that: (1) it is fast and easy to use; (2) it can be fully automated for online procedure; (3) it reduces the solvent and sample volume, as well as the waste produces; and (4) the cost of analysis is minimal when compared to conventional SPE. Overall, it is one of the most user- and environmental-friendly METs available for sample extraction.

MEPS is a very simple and straightforward MET, but it nevertheless involves a wide range of optimization steps that allow for a fine tuning of the extraction efficiency. 

#### 2.2.1. MEPS Influencing Parameters

##### 2.2.1.1. Sampling

Biological fluids, like urine, blood and plasma, are complex samples and should be processed accordingly to optimize the extraction of the target analytes by favoring a better interaction between sample analytes and the sorbent [35]. This involves the dilution of the sample (to reduce the sample viscosity), pH adjustment (to reduce the ionization of weak acids and bases for reversed-phase extraction), deproteination (with previous protein precipitation, for instance) and sample loading speed adjustment (an option in semi-automatic and automatic MEPS (reviewed in [9,35]).

##### 2.2.1.2. Number of Extraction Cycles (Draw-Eject)

In MEPS, the sample can be drawn through the needle into the syringe, once or several times (draw-eject), leading to a higher recovery level that should be optimized for each application. The multiple extraction cycles can be made from the same aliquot (draw-eject in the same vial) or by drawing up from an aliquot and discarding as waste (extract-discard) [35].

##### 2.2.1.3. Sorbent Type

This is probably one of the most important parameter to get high extraction recoveries in MEPS. There are nowadays many sorbent types available, from silica to polymeric and mixed-mode phases, functionalized or not, and even a porous graphitic carbon sorbent. In a simplified way, silica C2–C18 phases are more suitable for lipophilic analytes (non-polar) and polymeric phases, such as polystyrene-divinylbenzene or mixed-mode phases (anion-cation exchange mode), are more indicated for polar analytes such as acidic and basic compounds (reviewed in [9,35]). Nevertheless, in a significantly number of reports, custom sorbents, mainly molecular imprintings (MIPs) of the target analytes, have been successfully developed. Using this MIMEPS approach (MEPS using custom MIPs sorbents [9]), a higher analytical performance can be obtained in the following analytical procedures, and therefore, this format will certainly become more popular as they start to be commercially available.

##### 2.2.1.4. Washing Solution

In this step, unwanted and weakly retained interferents can be washed away. The solvent concentration and the pH are important factors to decrease the leaking of the target analytes under the washing process. It was shown, for instance, that the analyte leakage increased as the solvent percentage in the washing solution increases [35].

##### 2.2.1.5. Elution Solution

The elution solution should be an organic solvent, like methanol, isopropanol or acetonitrile, pure or mixed with acid or base solutions (0.1%–3%), and should be able to displace all analytes from the sorbent in a small volume (20–50 µL). Moreover, the solvent and the pH of the elution solution have a large influence on the recovery efficiency. The analyte elution increases as the solvent percentage and elution volume increase [35]. However, the best elution solvent should elute the maximum amount of analyte using the smallest volume possible, therefore increasing the target analyte concentration.

**Figure 4:**
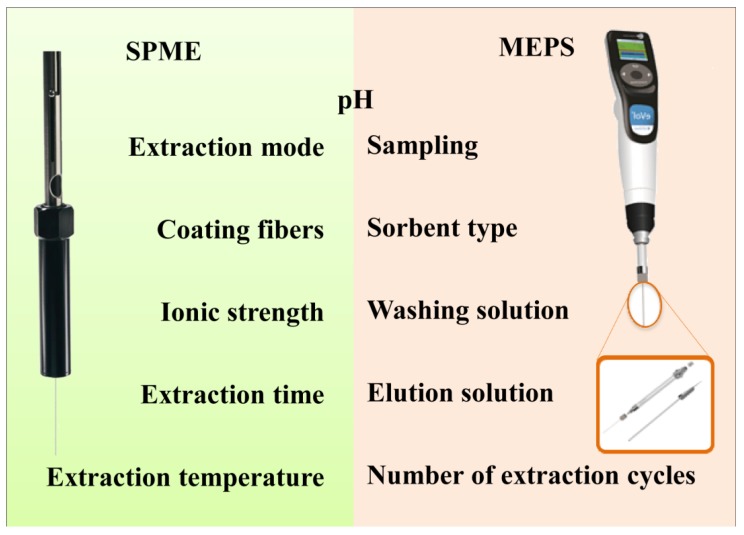
Most influent parameters in MEPS and SPME optimization.

Overall, as represented in Figure 4, MEPS and SPME present several optimization opportunities that should be carefully explored in order to improve the extraction of the target analytes.

Miniaturized analytical techniques had gained attention due to their many special features over conventional approaches. Among many advantages, the usage of little or no solvent, the low volumes of the sample required, the greater sensitivity in the sample preparation than for the exhaustive extraction procedures, the increasing of the sensitivity of analysis and a user-friendly system should be pointed out (Table 1).

**Table 1:** A comparison of some characteristics of target sample preparation techniques with solid phase extraction procedures. Reviewed in [11,36].

Factor	MEPS	SPE	SPME
Sorbent amount	0.5–4 mg	50–2,000 mg	150 mm Thickness
Sample preparation time	1–2 min	10–15 min	10–40 min
BIN (Barrel insert and needle) use	40 to 100 extractions	Single use	50–100 extractions
Sample throughput	low	high	high
Recovery	good	good	low
Sensitivity	good	good	low
Carryover	low	high	high
Cost	low	high	high

## 3. Metabolic Profiling of Urine: Recent Trends

Metabolomics can be defined as a method to assess the metabolite complexity of a given sample, and it involves the quantification of all metabolites in that sample. This approach allows for the evaluation of an extensive range of endogenous and exogenous metabolites that have an impact on the investigation of the physiologic status, the identification of biomarkers, the biochemical pathways that have been altered and the diagnosis of various diseases. The small-molecule metabolites are important in biologic systems and are potential candidates to understand disease phenotypes. These metabolites constitutes a wide group of low molecular weight compounds that include lipids, peptides, amino acids, nucleic acids, organic acids, carbohydrates, thiols, vitamins, *etc.*, and their characterization allows for obtaining unique “fingerprints” [37,38]. This provides an outstanding tool to assist disease diagnosis and prognosis and to identify new therapeutic targets and treatments. Additionally, metabolomics is also an important tool for many other applications, such as toxicology, environmental stress, nutrition, effects of genetic manipulation, identification of natural products, monitoring of donor organ viability and the establishment of genomic-metabolomic correlations. Recently, metabolomics has been focused on the identification of biomarkers for several diseases, namely cardiovascular (CVDs), oncologic (ODs) and neurodegenerative diseases (NDDs) [37,38,39].

In the course of history, urine has been a significant marker of health and illness. Hippocrates (460–355 BC) began using urine as a tool to diagnose disease and hypothesized that urine was a filtrate of the humors. Galen (AD 129–200) defined urine as a filtrate from blood and began to use it to characterize diseases that are recognized as diabetes mellitus and renal failure. Nowadays, urine analysis (urinalysis) has been well established as a diagnostic tool [39].

**Figure 5:**
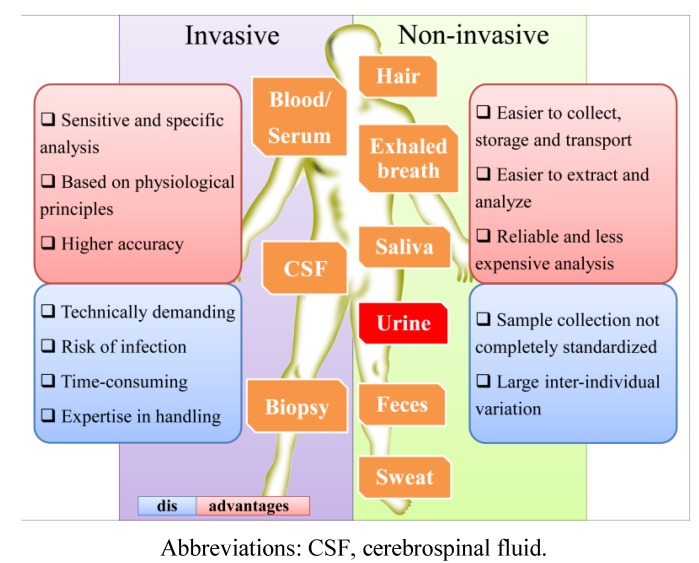
Comparison of the main aspects of the most common biological matrices.

Abbreviations: CSF, cerebrospinal fluid.

The use of urine as an analytical tool presents more advantages over other biological samples, such as blood, serum, plasma or tissue, since it can be obtained in large amounts by non-invasive sampling, requires no patient preparation and can be performed as often as needed [40,41]. Furthermore, in comparison with other biological fluids (Figure 5), urine presents lower protein content and sample complexity (mainly constituted by water, ions, creatinine and urea), including less intermolecular interaction, and thus, less sample pre-treatment is necessary [37,38,39,42]. As end products of normal and pathologic cellular processes, the metabolites present in urine are closely linked to phenotypes. Due to this, many efforts have been made over the last 10 years to study urinary metabolomics and to use it as a diagnostic tool [43]. Urinalysis became an excellent tool to obtain information about metabolite concentrations and pathways, errors in the normal metabolism, drug interactions and therapeutic drug monitoring (TDM), exposure to exogenous chemicals, drug abuse, particularly the illicit ones, doping control, nutrition and dietary intake, including food contaminants (reviewed in Zhang *et al.* [38]). For example, after soya consumption, daidzein, genistein and total isoflavonoids were found to be higher in urine samples [44], but in turn, the same urinary levels of genistein, total isoflavones and metabolites were reduced during antibiotic use [39]. For the development of specific biomarkers, it is important to decipher the complex molecular networks that characterize disease states [38]. For instance, the diagnostics methods that are usually employed are incapable of predicting jaundice syndrome (JS) in patients with liver disease. However, the results of one metabolomic study clearly revealed the potential of urine metabolomics in the diagnosis of this pathophysiology [45]. Urinary metabolites may be also very important for the non-invasive screening of pre-diabetic subtypes, which could lead to an early and personalized intervention for this disease [38]. Aiming for the characterization of metabolic indicators for type 2 diabetes mellitus (T2DM), Temmerman *et al*. [46] used LC-MS, GC-MS and NMR data from urinalysis to report a cross-platform for the urinary metabolome of T2DM. This allowed them to show significant up and down variations in the levels of several metabolites under hyperglycemia in T2DM.

Urinalysis has been also used to search for metabolite signatures that could be associated with cancer. In this way, several studies showed that low molecular weight urinary volatile organic compounds (VOCs) could be used as biomarkers for lung cancer, urine metabolomics having an excellent sensitivity (93%) and specificity (94%). Some metabolites of interest included β-hydroxyisovalerate, hippurate, α-hydroxyisobutyrate and creatinine [38,47].

Regarding the high prevalency breast and ovarian cancers, some qualitative and quantitative metabolomic methodologies have shown promising metabolic changes. Slupsky *et al.* [48], for instance, using NMR urinalysis, reported that intermediates of the tricarboxylic acid cycle and metabolites related with energy metabolism, amino acids and gut microbial metabolism were perturbed. This data is in agreement with the report from Serkova *et al.* [49], showing that several metabolic biomarkers related to glycolysis, mitochondrial citric acid cycle, choline and fatty acid metabolism are important in carcinogenesis and are responsive to anticancer treatment. Regarding this, Cho *et al.* [50] performed a targeted metabolite profiling of 14 urinary nucleosides in pre- and post-operative breast cancer female patients. The results showed levels of modified nucleosides (5-hydroxymethyl-2′-deoxyuridine, 8-hydroxy-2' -deoxyguanosine (8-OHdG), 1-methyladenosine and N(2),N(2)-dimethylguanosine) as being significantly higher in pre-operative patients than in both normal controls and post-operative patients. In the same direction, Loft *et al.* [51] observed a positive association between the urinary level of the oxidative stress biomarker, 8-OHdG, and the risk of especially estrogen receptor-positive breast cancers. Finally, Nam *et al.* [52] reported that urinary levels of homovanillate, 4-hydroxyphenylacetate, 5-hydroxyindoleacetate and urea were also altered in breast cancer patients.

Acute kidney injury (AKI) is another extensively studied clinical perturbation, due to its high incidence, mortality and morbidity. This justifies the search for suitable biomarkers, as the existing one, the monitoring of serum creatinine, presents poor analytical performance, being unspecific for AKI and highly influenced by other variables, such as gender, age, muscle mass and metabolism, drugs and hydration. Furthermore serum creatinine is not targeted for the early detection of disease [37]. Therefore, urine became a preferred matrix to search for new biomarkers for renal injury. Regarding this, Kim *et al.* [53] studied the potential of urine metabolomics to find important metabolites in kidney cancer and reported that some compounds related with amino acid and energy metabolism, such as quinolate, 4-hydroxybenzoate and gentisate, were differentially expressed [38].

Taylor *et al.* [54] performed a GC-MS metabolomics urinalysis of juvenile cystic mice to study kidney disease and found several changes in the purine and galactose metabolism pathways. In this report, allantoic acid and adenosine were identified as putative biomarkers of kidney disease that can now be studied in human urine. McClay *et al.* [55] performed NMR urine metabolomics to investigate the metabolic profiles of patients with chronic obstructive pulmonary disease (COPD) and showed that this approach was extremely useful for its diagnosis [37,55]. Michell *et al.* [56] studied Parkinson’s disease (PD) using a GC-TOFMS (Time-of-flight mass spectrometry) urine and serum profiling and observed changes in the urinary metabolite profiles that could be used for its diagnosis. However, the signatures obtained are still highly multivariated, and further experiments are necessary to assign specific urinary biomarkers for PD. Asthma was also studied using an untargeted metabolic approach for urine samples, and the profiles obtained revealed a potential to characterize the disease and to determine metabolites that may have a role in the principal pathogenic mechanisms [57,58,59]. The metabolites detected by Jung *et al*. [60] in patients that suffered from strokes showed that the urinary metabolomic approach may also be useful for the diagnosis of stroke pathogenesis. These examples show that urine metabolomics has a diagnostic potential for several disorders that should be improved to overcome certain problems, such as the high biological variation in its composition, the standardization of urinary metabolomics procedures and the validations of previous reports.

### 3.1. MEPS and SPME Advantages for the Metabolic Profile of Urine

In the previous section, a large number of selected applications showed the potentialities of the metabolic profile of urine in unveiling many diseases processes and pointing to new directions and their treatments. In most cases, however, instead of specific metabolites, we have information about up- or down-regulated pathways, but this information is too complex to be used in the clinical disease diagnostics. Furthermore, to make this information biochemically relevant, it would require long and complex analysis. Therefore, these metabolic profiles should instead provide biomarkers able to be readily used in disease biomarker. In this sense, SPME and MEPS are suitable for fulfilling this objective of breaking urine metabolomic complexity. Different high throughput solutions are nowadays available to easily process a large amount of samples, speeding up metabolomic research. Several applications in different fields of research have been reviewed [9,61,62], but here, we will focus in more detail on specific applications to the most prevalent human diseases, Cardiovascular (CVDs), oncologic (ODs) and neurodegenerative diseases (NDDs).

### 3.2. Early Diagnosis of Highly Prevalent Diseases, Such As Cardiovascular, Oncologic and Neurodegenerative Ones

CVDs, ODs and NDDs are the three main causes of mortality worldwide. Moreover, the morbidity burden associated with these diseases is also very high, presenting an increasing trend that is proportional to the rise in life expectancy. The main cause of this scenario is the late diagnosis of these diseases, which, moreover, is usually performed in invasive and expensive formats, when the diseases already have reached the medium to late stages. Therefore, there is a great potential to unveil using urine as a tool to achieve an early and potentially inexpensive disease diagnose. Although these diseases develop slowly and progress in a relative asymptomatic way till they reach an advanced stage development, there are certainly causes and modifications that can be assessed in order to obtain an early diagnosis, particularly specific metabolites, whose levels are necessarily different from the healthy status. Oxidative damage to the cell components (particularly lipids of the membrane layer, proteins and nucleic acids) caused by reactive oxygen and nitrogen species (ROS and RNS, respectively) is nowadays recognized as a hallmark in these diseases. In fact, several reports point to oxidative damage as a triggering event in different ODs, NDDs and CVDs [63,64,65,66]. CVD progression, for instance, is characterized by an increase in lipid peroxidation that causes vascular lesions, inflammation events and, finally, atherosclerosis [67,68]. Therefore, there should be different metabolites that reflect these events, these being potentially suitable biomarkers for the early diagnosis of CVD progression. The success of this approach depends, however, on powerful analytical methodologies able to reliably characterize metabolites in vestigial concentrations. In the last few years, improvements in liquid chromatography (LC), gas chromatography (GC) and mass spectrometry (MS) have allowed an unprecedented analytical performance, able to go deeper into the characterization on new disease biomarkers. If properly conjugated with microextraction techniques (METs), namely MEPS and SPME, the limit of the single molecule quantification is increasingly closer. This fulfills the metabolomic characterization of the main human diseases that is necessary to find the best biomarkers for an early diagnosis. Additionally, the powerful analytical performance that can be obtained by using high throughput platforms conjugating METs with LC, GC and MS is being successfully used in the characterization of a vast number of drugs and drugs metabolites used against diseases (reviewed in [13,61,62]). This allows for a sophisticated therapeutic drug monitoring that is critical for the success of the treatment of many diseases, particularly CVDs, OD and NDDs [69]. Several relevant reports on this subject are described in Table 2. Additionally, some applications using SPE will be also highlighted, which would greatly benefit from an upgrade to the MEPS methodology.

**Table 2:** Representative reports using MEPS and SPME methodologies in the characterization of target urinary metabolites.

Target diseases or analytes	LOD	LOQ	Analytical method	Reference
	(ng·mL^−1^ by default)			
**CVDs**				
F2-Isoprostanes (oxidative damage biomarker)	-	-	SPE/LC-MS	[70,71,72,73]
	-	-	SPE/GC-MS	[74,75]
a-KG, L-CAR and acetyl-L-CAR (cardiac cell metabolism)	-	0.04–0.08	SPME/LC-MS	[76]
**CVDs drugs**				
Aliskiren, prasugrel and rivaroxaban	-	0.5–5.0 pg·mL^−1^	MEPS/LC-MS/MS	[77]
Pravastatin and pravastatin lactone	1.5 nM	5 nM	SPME/LC-MS	[78]
Stimulants and β-blockers	0.1–1.2	-	SPME/LC-MS	[79]
Acebutolol and metoprolol	-	1.0	MEPS/LC-MS/MS	[80]
Propranolol	4–7	13–20	SPME/CEC	[81]
Verapamil, propranolol and metoprolol	-	-	MEPS/µPESI-MS/MS	[82]
Verapamil, gallopamil, norverapamil	52–63, 5–8	-	SPME/LC-UV (LC-MS)	[83]
Lidocaine	1.0	5.0	MEPS/LC-MS/MS	[36,84]
ODs				
Hexanal and heptanal (lung cancer)	0.10–0.11	0.21–0.23	SPME/GC-MS	[85]
Breast cancer VOCs	-	-		[41]
Prostate cancer VOCs	0.10	0.16		[86]
Sarcosine and N-ethylglycine	-	0.03–0.06		[87]
Neuroendocrine tumor markers (HVA, VMA, 5-HIAA)	0.046–24.3	0.063–49.6	SPME/GC-QqQ-MS	[88]
Unrelated cancer forms	-	-	SPME/GC-MS	[89]
5-HMUra and 8-oxodG (oxidatively damaged DNA)	0.05–4.0	0.23–130.0	MEPS/LC-PDA	[90]
8-hydroxy-2′-deoxyguanosine (oxidatively damaged DNA)	2.04 nM	7.12 nM	SPME/LC-UV	[91]
	2.61 nM	8.63 nM	SPME/CE-ECD	[92]
**ODs**				
17β-estradiol and 2-methoxyestradiol (potential angiogenesis modulators)	-	-	SPME/GC–MS	[93]
Roscovitine (potential anticancer drug)	0.5	1.0	MEPS/LC-MS/MS	[94]
Olomoucine (potential anticancer drug)	0.5	1.0	MEPS/LC-MS/MS	[95]
Acrolein (lipid peroxidation by-product and metabolite of cyclophosphamide and ifosfamide (anticancer drugs))	-	-	SPME/GC-MS	[96]
**NDDs**				
Selegiline and desmethylselegiline (PD drugs)	0.01–0.05	0.05–20	SPME/GC–MS	[97]
**Catecholamines (elevated in several CVDs an NDDs)**				
Serotonin, dopamine and noradrenaline	2–20	5–50	MEPS/LC-ECD	[98]
Dopamine and serotonin	1	50	MEPS/LC-MS/MS	[99]
Dopamine	1.2	4.0	SPME/LC-ESI-MS/MS	[100]
Dopamine, epinephrine and norepinephrine	4.8–7.4 (nM)	-	SPME/EC-UV	[101]
non-polar heterocyclic amines (high carcinogenic potential)	1.6–5.6	5.5–18.7	MEPS/CLC-FLD	[102]

Abbreviations: 5-HMUra, 5-hydroxymethyluracil; 5-HIAA, 5-hydroxyindolacetic acid 8-oxodG, 8-oxo-7,8-dihydro-2′-deoxyguanosine; AD, Alzheimer’s disease; ALS, amyotrophic lateral sclerosis; CAR, carnitine; CEC, Capillary electrochromatography, CLC-FLD, capillary liquid chromatography - fluorometric detection; CVDs, cardiovascular diseases; GC-MS, gas chromatography-mass spectrometry, GC-QqQ-MS/MS, gas chromatography-triple quadrupole-mass spectrometry; HPLC-PDA, high performance liquid chromatography with photodiode array detection; HVA, homovanillic acid; KG, ketoglutaric acid, LC-ED, high performance liquid chromatography coupled to electrochemical detection; LC-ESI/MS, liquid chromatography coupled to an electrospray ionization mass spectrometer; LC-CD, liquid chromatography with colorimetric detection; LC-FLD, liquid chromatography with fluorescence detection; LC-MS/MS, liquid chromatography-tandem mass spectrometry; LC-UV, liquid chromatography coupled to an ultraviolet detector; LOD, limit of detection; LOQ, limit of quantification; METs, microextraction techniques; NDDs, neurodegenerative diseases; ODs, oncologic diseases; PD, Parkinson’s disease; SPME, solid-phase microextraction; µPESI-MS/MS, micropillar array electrospray ionization mass spectrometry; VMA, vanilmandelic acid; VOCs, volatile organic compounds.

#### 3.2.1. CVDs

CVDs designate several heterogeneous circulation disorders that include rheumatic, hypertensive, ischemic, cerebrovascular and inflammatory heart diseases. These conditions are primarily caused by an early and asymptomatical atherosclerosis that progresses gradually through adolescence and early adulthood [103]. Their diagnose is still nowadays performed through the widely conventional risk factors associated with the onset of CVDs, such as high blood pressure, hypercholesterolemia, age, gender, obesity, life style abuses, *etc*. However, these parameters are not suitable for an early diagnostic of CVD progression, at least if not conjugated with other biochemical biomarkers. Moreover, they are also not able to predict sudden and fatal occurrences of CVDs, such as acute myocardial infarctions and strokes. In this way, there are interesting reports pointing to other directions, particularly to oxidative damage biomarkers. F2-isoprostanes and 8-OHdG are generally accepted as biomarkers for monitoring oxidative status over time [104], and in fact, there are several reports suggesting F2-isoprostanes as prognostic CVD biomarkers ([105] and the references within [106,107]). F2-isoprostanes, which are generated by the free radical-mediated peroxidation of arachidonic acid, a key component of the cell membrane, are mainly quantified by LC-MS or GC-MS following an SPE extraction [70,71,72,73,74,75,104,108] or ELISA, although the reliability of this approach is strongly affected by antibody cross-reactivity issues [109]. The utilization of MEPS would certainly raise the analytical performance obtained in the methods described, allowing for a deeper understanding of the power of F2-isoprostanes as CVD biomarkers. Using this extraction procedure, Mendes *et al.* [110] reported another putative correlation between oxidative damage and CVDs. Although very preliminary, their results suggest a positive correlation between the urinary levels of the oxidatively damaged DNA adducts, 8-oxo-7,8-dihydro-29-deoxyguanosine (8-oxodG) and 5-hydroxymethyluracil (5-HMUra) and CVDs risk progression. Magiera *et al.* [76], in turn, focus their studies on α-ketoglutaric acid (KG), L-carnitine (CAR) and acetyl-L-CAR, which are molecules that have an important role in cardiac cell metabolism, this being very important for the outcome of a cardiac injury. In this case, the authors used a two-step SPE procedure using silica gel and quaternary amine cartridges for sample cleanup, which limits the high throughput potential of the methodology. A large number of drugs used for different CVD etiologies are available nowadays, raising questions about the possible side effects and drug interactions. As pointed out by Baranowska *et al.* [111], several studies show that the most common causes of adverse events in a hospitalized patients review are related to cardiovascular drug treatments. Therefore, the existence of reliable methodologies to follow the effects of these drugs and their metabolites in the organism is very pertinent. To this point, we should highlight among the several examples shown in Table 2 the first reports for the quantification of urinary and plasmatic β-blockers using automated in-tube SPME coupled with LC/ESI-MS [112] and with LC-UV [113]. These drugs are widely used for the treatment of several CVDs (hypertension, angina pectoris and cardioarrhythmias), but they are very toxic and their therapeutic ranges very narrow [112]. In another report, El-Beqqali *et al.* [80] combined MEPS and LC–MS/MS to determine urinary acebutolol and metoprolol, β-adrenoceptor-blocking drugs used as anti-hypertensive and anti-anginal agents. Furthermore, the application of the same methodology for the quantification of the very recent and promising cardiac drugs aliskiren, prasugrel and rivaroxaban, is very relevant [77].

#### 3.2.2. Oncologic Diseases (ODs)

ODs are complex diseases triggered by different conditions and circumstances, but inevitably start with a cell that lost its normal homeostasis and started proliferating without control. These abnormal cells possess dramatically affected metabolisms, particularly at the glycolysis level (the “Warburg effect”), which they use to produce the amino acids, nucleotides and lipids necessary for their proliferation [114]. ODs will therefore produce changes that can be measured, whether they are changes in the abundance of certain compounds or new metabolites. Regarding this, the application of SPME to identify VOCs is a popular approach and has been successfully applied mainly to the characterization of the exhaled breath of cancer patients, particularly lung and breast cancer (reviewed in Pereira *et al.* [13]), but also urine. This is supported by several experimental observations, particularly the olfactory detection of prostate cancer by dogs sniffing urine [115]. Further evidences were obtained using SPME-GC/MS to characterize the urinary VOCs signatures for different forms of cancer, including lung [85], breast [40], lymphoma, leukemia and colorectal cancer [89] and prostate [86]. In most of these cases, a relevant part of the VOCs signatures is caused by oxidative damage, as in the case of the aliphatic hydrocarbons, such as the aldehydes ethane and pentane, which are the main by-products of lipid peroxidation and oxidative DNA damage. This observation is supported by epidemiological evidence that clearly points to oxidative damage as one of the major sources of carcinogenesis [116]. However, as highlighted by Kwak and Preti [117], although some urinary disease-associated VOCs have been used in clinics, more experimental work is necessary to consistently demonstrate the strength of some of those applications. Regarding this, Monteleone *et al.* [88] developed a fast SPME/GC-QqQ (triple quadrupole)-MS methodology to quantify three catecholamines metabolites (homovanillic acid (HMA), vanilmandelic acid (VMA) and 5-hydroxyindoleacetic acid (5-HIAA)). Catecholamines, particularly noradrenaline, adrenaline and dopamine, are important neurotransmitters in the sympathetic nervous system, exerting cardiovascular and metabolic effects through adrenergic receptors stimulation in a wide variety of cells [118]. Their quantification is used in the diagnosis and management of patients with neurocrine tumors, such as phaeochromocytoma, and therefore, the application of this methodology in the clinical environment will have a major impact on the early diagnosis, disease progression and, in many cases, drug concentration, enabling important therapeutic drug monitoring (TDM) [13]. Additional works reporting the quantification of these catecholamines using MEPS and SPME are presented in Table 2 [98,99,100]. In another direction, Mendes *et al.* [90] used a fast and simple MEPS/LC-UV methodology to analyze the presence of oxidatively damaged DNA molecules in urine and reported that 8-oxodG levels are significantly augmented in breast and lung patients, while 5-HMUra is clearly less abundant in the urine from lung cancer patients in comparison to breast cancer and control samples. In turn, Barocas *et al.* [119] reported that oxidative stress measured by urinary F2-isoprostane levels is associated with prostate cancer. This work, performed in an SPE/GC-MS format, shows the potential of F2-isoprostanes as biomarkers of prostate cancer. A microextraction approach applied to other ODs is therefore very pertinent and certainly will clarify if F2-isoprostane can be used as an OD biomarker.

#### 3.2.3. Neurodegenerative Diseases (NDDs)

The accumulation of oxidative damage has been implicated in aging and various neurological disorders, including Alzheimer’s disease (AD), Parkinson’s disease (PD) and amyotrophic lateral sclerosis (ALS) [120,121]. Therefore, the search for urinary biomarkers of NDDs was primarily focused on oxidative damage biomarkers. To this point, Bolner *et al.* [122] showed that 8-OHdG alone, as well as the ratio 8-OHdG/2-dG (the corresponding non-hydroxylated base, 2′-deoxyguanosine) were significantly different in healthy controls and Parkinson disease (PD) patients. Similarly, Bogdanov *et al.* [123] reported increased levels of free 8-OHdG in urine, plasma and cerebrospinal fluid (CSF) of ALS patients. These is, therefore, important evidence that support the importance of 8-OHdG as a biomarker to complement NDDs diagnosis, and a faster methodology using MEPS/UHPLC-UV has been already described [90,110]. Regarding F2-isoprostanes as biomarkers of oxidative stress, unlike the CVDs ([105] and references within, [106,107]) and ODs [119], there are conflicting results about their role as biomarkers in different NDDs, such as AD, PD, ALS or Huntington’s diseases [124,125,126,127,128,129,130]. This inconsistency is probably due to the inability of some of the methodologies developed to differentiate between the several isoprostane isoforms, originating misleading results that need to be clarified with further studies. However, as there is a clear increase in the lipoperoxidation products, malondialdehyde (MDA) and 4-hydroxy-2-nonenal (4-HNE) in fibroblasts and lymphoblasts of familial AD [131], it is very plausible that isoprostanes, at least some isoforms, also by-products of lipid peroxidation, are increased in most NDDs. As shown by Cecchi *et al.* [131], MDA quantification in urine is a promising opportunity to improve NDDs diagnosis, and a fast and reliable MEPS/UHPLC-UV methodology for the simultaneous quantification of MDA and 8-OHdG has been already described [90,110]. Finally, Davidson *et al.* [132] showed that PD patients under L-dopa treatment present significantly higher values for urinary dopamine, homovanillic acid, free normetadrenaline and free metadrenaline. L-dopa is a precursor of dopamine and other catecholamines and an important antiparkinsonian drug. Therefore, this is an interesting TDM application that could be improved by the utilization of fast MEPS or SPME strategies, such as the ones reported for dopamine quantification [98,99,100].

## 4. Concluding Remarks

Urine is a biological matrix with a great potential for the early diagnosis of the highly prevalent CVDs, ODs and NDDs that has not yet been fully explored.

**Figure 6:**
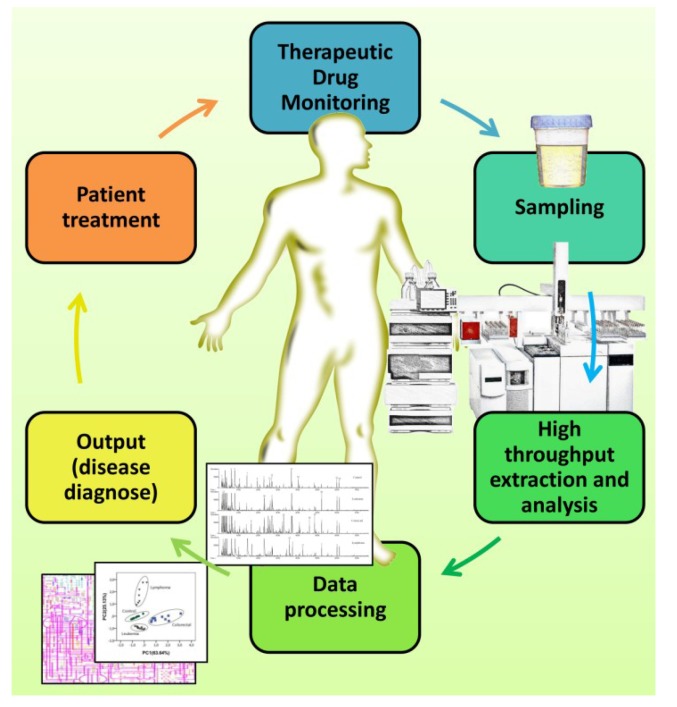
Flowchart for the SPME and MEPS high throughput potential applied to urinalysis for the metabolic profiling of urine and the early diagnosis of high prevalent diseases.

The major reason for this potential is that human metabolism under disease conditions is necessarily different from the steady state, producing changes in the presence and abundance of specific metabolites. In this way, metabolomics is a growing and powerful technology capable of detecting hundreds to thousands of metabolites in tissues and biofluids (Figure 6). These metabolites, if properly identified, may contribute to a faster, reliable and non-invasive method of diagnosis. Urine has not been fully explored as an early diagnosis method, and its complexity (it contains, at least, 3,079 detectable metabolites, as Bouatra *et al.* [133] very recently reported in the human urine metabolome database) and the analytical difficulties in identifying and quantifying vestigial metabolites are certainly the two main reasons accounting for that fact. Microextraction techniques, such as SPME and MEPS, can be successfully used to simplify this complexity and improve the analytical performance of the LC, GC and MS available nowadays even more, allowing for the characterization of the metabolite profile, which can be reliably used for an early diagnosis of a given disease, namely the CVDs, ODs and NDDs analyzed here.

One of the future aspects of SPME and MEPS for clinical bioanalysis by metabolomics profiling studies would be using *in vivo* SPME models, such as humans, rather than in rats or dogs, as they are performed at the moment (reviewed in [13]). This kind of application would be very interesting once the information obtained is more accurate and in the real-time functioning of the organism. This approach would reduce contact with biological fluids and contamination issues. Regarding MEPS, it would be interesting to study the potential of this extraction technique in metabolites from urine, as described in the previous section, as a promising fluid for the screening of diseases.
